# Does Muscle Development of Sport Horses Using Water Treadmill Exercise as Part of a Long-Term Training Programme Differ from That of Horses Not Using Water Treadmill Exercise?

**DOI:** 10.3390/ani15162426

**Published:** 2025-08-19

**Authors:** Carolyne Tranquille, Kathryn Nankervis, Jack Tacey, Emily Hopkins, Isabeau Deckers, Vicki Walker, Russell MacKechnie-Guire, Richard Newton, Rachel Murray

**Affiliations:** 1 Equestrian Performance Research Centre, Hartpury University, Hartpury, Gloucester GL19 3BE, UK; kathryn.nankervis@hartpury.ac.uk (K.N.); victoria.walker@hartpury.ac.uk (V.W.); russell.mackechnie-guire@hartpury.ac.uk (R.M.-G.); 2 JBT Veterinary Physiotherapy, Nottingham NG10 4EH, UK; jbtvetphysio@gmail.com; 3 Regain Veterinary Physiotherapy, Bury St Edmunds IP31 3SL, UK; eghopkins@outlook.com; 4 Equilibro, Foxemaatstraat 27, 2920 Kalmthout, Belgium; info@equi-libro.com; 5 Department of Medicine, University of Cambridge, Cambridge CB3 0ES, UK; rn428@cam.ac.uk; 6 Ibikus Ltd., Bury St Edmunds IP32 7AR, UK

**Keywords:** equine, hydrotherapy, muscle development, body condition score

## Abstract

Water treadmill (WT) exercise has become a popular tool for equine training and rehabilitation. However, few studies describe the long-term effects of WT exercise in low water on muscle development (MD). This study’s objectives were to compare MD changes over a 40-week period in sport horses that regularly used WT in low water within training (Group WTH, *n* = 55) and a control group that did not (control, *n* = 28). Subjective MD assessments were undertaken using an adaptation of a previously published method at weeks 0, 20 and 40. For Group WTH, MD significantly increased in the neck, pelvis and hindlimb adductor and abductors between weeks 0 and 20. Neck, thoracic, thoracic trapezius, lumbar, pelvis, quadriceps, hindlimb adductor and abductor, and hamstring musculature increased between weeks 0 and 40. Thoracic musculature only increased between weeks 20 and 40 (*p* ≤ 0.001 for all). In the control horses, MD did not significantly change between weeks. Regular WT exercise appears to increase MD, particularly for musculature used to create movement patterns seen on the WT. WTs may be appropriate for use under veterinary guidance as part of a directed rehabilitation/training programme to increase core and hindlimb MD.

## Simple Summary

There is increasing interest in the long-term effect of water treadmill exercise on muscle development in horses. We aimed to describe and compare changes in muscle development in a group of horses that regularly use a water treadmill and in horses that do not. To achieve this, we assessed muscle development at 20-week intervals, using an adaptation of a previously published grading scale, over a 40-week period. Fifty-five horses that used the water treadmill regularly and twenty-eight horses that did not use the water treadmill were recruited. In the horses that regularly used the water treadmill, we found that muscle development significantly increased over time in all areas assessed. For some muscle groups, changes were only observed at the end of the 40-week period. The biggest changes were seen in the hindlimb musculature. No changes were seen in the horses that did not use the water treadmill. Water treadmill exercise appears to increase the development of muscles that support movement in water.

## 1. Introduction

The plasticity of equine skeletal muscle and the nature of its generic responses to training programmes are reasonably well understood [1,2,3]. The ability to modulate adaptive responses in accordance with the type of training programme applied appears possible, including endurance, strength and speed [4,5,6,7]. Much work is directed towards trying to elucidate the optimal training stimuli to elicit the most appropriate muscular responses for improvement in performance in any given sport. Positive adaptive responses are initiated by inclusion of exercise specific to the sport in question, whilst taking care not to induce injury due to repetition and/or overload [7]. In human and equine training, cross-training or multimodal exercise is often advocated as a means of performance enhancement whilst avoiding overuse injury [8,9,10]. Exercise which supports competition performance through targeted sport-specific muscle adaptation, e.g., increased gluteal muscle strength, without replicating the precise physical demands of competition is therefore often sought. For example, hill work is often utilised to increase training intensity (and training stimulus) at any given speed, reducing the need for speed work and reducing the injury risk associated with speed work [11]. Water treadmill (WT) exercise provides a potentially beneficial form of low-intensity exercise for cross-training, with the properties of water (increased buoyancy, increased viscosity, hydrostatic pressure) conferring advantageous effects such as reduced impact shock [12], improved postural stability [13] and increased distal limb and back movement ranges [14,15,16,17,18,19]. WT exercise is now a popular tool for rehabilitation and training of horses [20,21,22], with owners choosing to use it for ‘increased strength’ and ‘general performance improvement’ amongst other reasons [21], although there is relatively little, but growing, evidence to date regarding the long-term effects of its use on equine muscle development (MD) and overground locomotion patterns.

Only a few studies to date have provided evidence that WT exercise can contribute to increased muscle mass in horses. Van de Winkel et al. [23] used transcutaneous ultrasound to measure changes in muscle cross-sectional area in five Friesian horses throughout eight weeks of aqua-training, for five days per week, 20 min per session, at 1.25 m/s and a water depth of 40 cm. WT exercise caused hypertrophy of specific muscles in the forelimb, back and hindlimb, particularly muscles involved in elevation and forward movement of the forelimb, i.e., brachiocephalicus and cervical and thoracic trapezius, thoracic erector spinae, and flexion within the hindlimb, i.e., quadriceps femoris and vastus lateralis, but only on the left-hand side. This evidence regarding hypertrophy of certain musculature was in contrast to previous studies which had found no evidence of changes in metabolic characteristics of the superficial digital flexor or gluteus medius muscles following four weeks of WT training [24] and no evidence of changes in muscle fibre properties or metabolic characteristics following eight weeks of WT training [25] in horses exercising up to 40 min per day, five days per week, in water level with the olecranon.

More recent studies have provided evidence that WT training can induce muscle hypertrophy in specific regions. Fair et al. [26] measured back muscle profiles throughout a four-week WT training programme incorporating WT exercise at metacarpal-depth water on a 4% incline for 15 min six days a week and found small, yet significant, increases in back muscle dimensions as measured using a flexible curve ruler to capture back muscle profiles. A flexible curve ruler method was also used by Rogge et al. [27] to measure changes in a larger group of horses (*n* = 15) undertaking WT exercise three times per week (depth not stated) for up to 30 min. These authors found increases in muscle dimensions at the level of the tenth thoracic vertebrae, but not at the fourteenth and eighteenth thoracic vertebrae or the third lumbar vertebrae, after nine weeks of training. However, neither of these studies compared WT training to a control. A study employing ultrasound to measure changes in muscle mass in horses trained on a WT (*n* = 7) and a dry treadmill (*n* = 5) found that eight weeks of WT training resulted in significant increases in muscles of the forelimb and hindlimb, but not of the back musculature examined (thoracic and lumbar erector spinae), in a group trained on the WT for 20 min per day, five times a week, at mid-metacarpal depth [28]. Horses trained on the dry treadmill at the same duration and frequency only showed increases in forelimb musculature. The majority of muscle mass changes occurred between weeks 4 and 8 of training. Subjective muscle scoring systems for assessment of MD based on a combination of visual assessment and palpation have previously been described for use in sport horses [29] and Thoroughbreds [30]. Whilst ultrasonography provides a reliable, objective method of measuring muscle mass [31], subjective visual assessment is still an attractive option for in-field measures due to speed and ease of application [30].

Although previous studies have investigated the effect of WT training alone on MD, this is less representative of the training pattern of most horses using the WT, where WT exercise forms only part of their training programme. It is therefore important to understand more about the effect of WT training as part of a sport horse training programme. It would be expected that horses undergoing WT exercise as the exclusive form of exercise would show faster changes in MD than horses using the modality at a much lower frequency, such as in horses using it as part of a normal training programme. The aim of this study was therefore to investigate muscle mass changes in a large group of sport horses using WT exercise in low water as part of their normal training programmes, and for a longer period of time than previously studied. We hypothesised the following: 1. Horses incorporating WT exercise in low water as part of their training regime will have higher MD scores of the hindlimbs and epaxial musculature than a matched control group trained without WT exercise. 2. In WT horses, the majority of the increase in MD scores will be evident between weeks 0 and 20 compared to weeks 20 and 40.

## 2. Materials and Methods

Ethics were approved by the Ethics Committee of the Animal Heath Trust (AHT 42-2017). Informed, written consent was obtained from the owners prior to participation, and they were advised that they could withdraw from the study at any point.

### 2.1. Sample Population

A sample of 55 WT-trained (11.1 ± 3.9 years) sport horses using WT training as a part of their overall training programme (Group WTH), and 28 work-matched control (Group C) sport horses (mean age: 11 ± 3.9 years) (Table 1) was recruited for the study. Group-WTH horses were recruited from two commercial WT venues with large client bases within the South of England. Group-C horses were recruited from three privately owned yards.

The sample size was determined based on the previously reported sample size necessary for the measurement of kinematic data [19]. MD was captured as part of this study using the same treatment (Group WTH) and control (Group C) horses. For all horses, week ‘0’ was between February 2019 and May 2019, week ‘20’ between July 2019 and September 2019 and week 40 between December 2019 and February 2020. Horses were divided into groups based on their location and availability, and data were collected at week 0 and at weeks 20 and 40. Testing days were staggered to enable data collection to occur across the two WT venues and three private yards.

All horses included in the study were deemed fit for participation in their chosen discipline with no recent history of lameness and were not under any investigations relating to orthopaedic disease or loss of performance. Soundness was verified by a gait evaluation based on an International Equestrian Federation pre-competition veterinary assessment by an orthopaedic specialist (RM), including straight-line walk-and-trot assessment at weeks 0, 20 and 40. Horses were included in the study if they were deemed sound (defined as a lameness grade of less than 1/10) [32].

All horses using the WT (Group WTH) were habituated to the WT preceding the start of the study and routinely used the WT (weekly *n* = 21, every two weeks *n* = 19, three times per month *n* = 5, more than once a week *n* = 10). Group-WTH horses’ experience varied at time ‘0’ as follows: less than five weeks of experience (*n* = 2), five to 24 weeks (*n* = 9), 25 to 52 weeks (*n* = 17), 53 to 78 weeks (*n* = 6) and 79 to 104 (*n* = 13), and group six had more than 104 (*n* = 8) weeks of experience. Control horses (Group C) had not previously used a WT and were broadly type- and exercise-matched to the WT horses.

At the start of the study, horse owners provided information on horse signalment, and work discipline and level, information on previous musculoskeletal injuries, duration of WT use and normal frequency of WT use, details of the most recent WT session and a summary of an average exercise plan for any given week. Horses were grouped according to their normal frequency of WT usage: three times per month, every two weeks, weekly and more than once a week. Over the 40 weeks, all horses (WT and controls) were required to continue with their general training and competitive programme as prescribed by the owners, riders and trainers. The WT training programme was defined on an individual basis by the WT venue and operators with input from the study veterinarian (RM) and WT specialist (KN) after each test session to enable the horse to progress over time. For some horses the water depth increased with time, and for others it did not, depending on their size, fitness and response to WT exercise. As with any carefully managed training programme, the horses’ sessions were not all the same during the study time. All horses were exercised at what is considered a low depth (i.e., below the tarsus). Horses were exercised in walk on the WT, and protocols were defined based on the following factors according to industry guidelines [33]: being able to maintain position in the centre of the belt, body posture being similar to overland walking posture, the head remaining largely still, maintaining a regular rhythm to the footfalls and the hindlimbs being placed in the path of the forelimbs. The industry guidelines [33] were created by an international group of researchers, academics, veterinary clinicians and practitioners, combining those involved in generating the evidence relating to WT exercise, those responsible for dissemination of the research and those who use WTs. The aim of the guidelines was to provide information on the optimal use of the modality within a training or rehabilitation programme of horses. Training sessions were no more than 25 min in duration, at a training speed of 3.6 kph to 5.4 kph (mode 4.6 kph), i.e., 1.0–1.5 m/s (mode 1.3 m/s), and at the following water depths: fetlock (*n* = 4), mid-cannon (*n* = 16), distal carpus (*n* = 5) and carpus (*n* = 30).

### 2.2. Muscle Development Scoring

**Figure 1:**
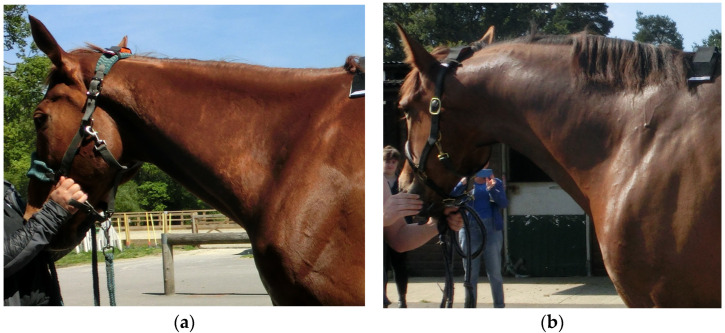
(**a**) This horse was given a Grade 1 for the development of the neck; (**b**) this horse was given a Grade 4 for the development of the neck. Note that these are different horses, not the same horse at different time points.

**Figure 2:**
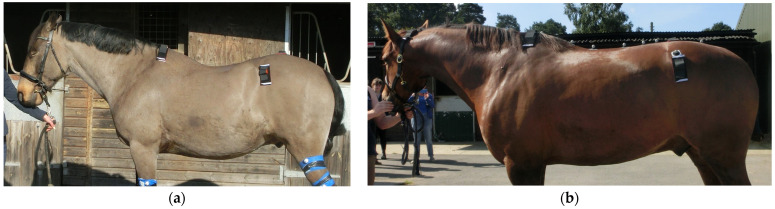
(**a**) This horse was given a Grade 1 for the development of the thoracic trapezius and abdominals; (**b**) this horse was given a Grade 3 for the development of the thoracic trapezius and Grade 4 for the development of the abdominals.

**Figure 3:**
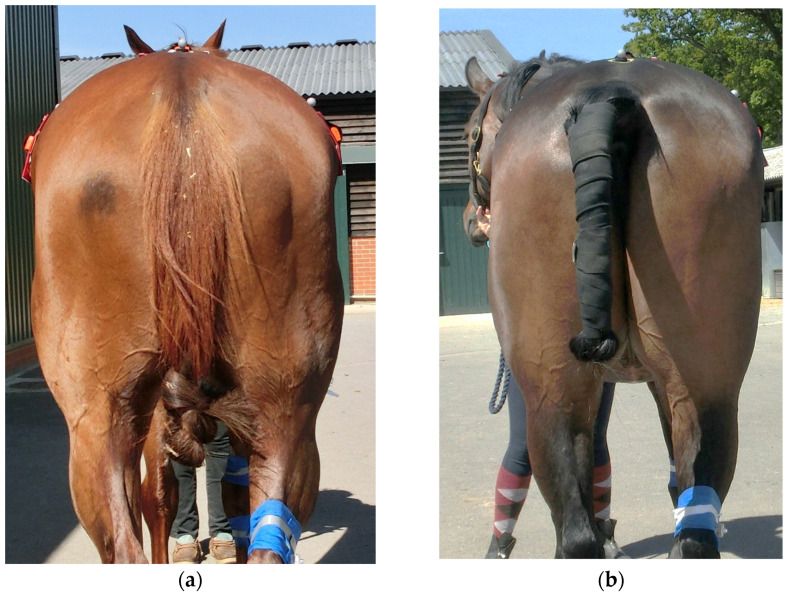
(**a**) This horse was given a Grade 2 for the development of the hamstrings and pelvis (gluteal) musculature; (**b**) this horse was given a Grade 4 for the development of the hamstrings and of the pelvic musculature.

Subjective MD grading assessment of specific regions was undertaken using an adapted version of a previously published 1–5 grading scale, with ‘5’ denoting greater MD mass with better muscle tone and response to palpation than ‘1’ [29] (Supplementary Table S1), to obtain scores for the following regions of musculature: ‘neck’ (Figure 1), ‘cervical trapezius’, ‘thoracic trapezius’ (Figure 2), ‘thoracic epaxial’, ‘lumbar epaxial’, ‘pelvis’ (gluteal musculature), ‘hamstrings’ (Figure 3), ‘quadriceps’ (including vastus lateralis) and ‘hindlimb abductors and adductors’, and ‘abdominal’ (Figure 2). For the current study, the cervical trapezius, thoracic trapezius, hamstring, quadriceps and hindlimb abductor and adductor musculature was added to the published grading system as a more detailed adaptation of it, yielding ten specific regions as opposed to just six [29]. The sum of the 10 locations was then used to create a total MD score for each time point for each horse.

MD assessment took place with the horses standing square on a level concrete surface prior to completing the WT test. Palpation was carried out with a flat hand in the direction of the hair and with light pressure. MD assessments were carried out by an experienced veterinarian (RM) and a trained research assistant (CT); inter- and intra-observer repeatability was confirmed prior to data collection [6]. The 9-point Henneke body condition score (BCS) system [34] was used to assess each horse at the same time as the MD assessment. Horses were grouped according to geographical location as a convenience sample, and assessments were carried out in a staggered manner to limit seasonal influence [35]. For repeated assessments at weeks 20 and 40, assessors were blinded to previous scores.

### 2.3. Statistical Analysis

Descriptive statistics were obtained for each variable in each condition using statistical analysis software (JASP version 14.1). MD grades for each region were compared between weeks 0, 20, and 40 using a Friedman test with a post hoc Wilcoxon signed-rank test. Control horses’ MD grades were compared between week 0 and week 20 using a Wilcoxon signed-rank test (SPPS version 29.0). One-way ANOVA was used to test the effect of frequency of WT usage on changes in MD score for each location between all time points. BCS for WTH horses was compared between weeks 0, 20, and 40 using a Friedman test with a post hoc Wilcoxon signed-rank test. Control horses’ total BCS was compared between week 0 and week 20 using a Wilcoxon signed-rank test (week 40 unavailable). Eighteen control horses were lost due to COVID-19 restrictions preventing reassessment, resulting in only five horses completing the 40-week period; hence, statistical analysis for weeks 0–40 was not conducted. An adjusted *p* value of *p* ≤ 0.001 was used due to multiple comparisons. A mixed-effect regression multivariable model, with horse treated as a random effect to control for repeated measures within each horse, was used to determine the effect of group (Group WTH vs. Group C), discipline and competition level on total MD score (Stata 15.0) with a significance value of *p* ≤ 0.05 applied to the model.

## 3. Results

**Figure 4:**
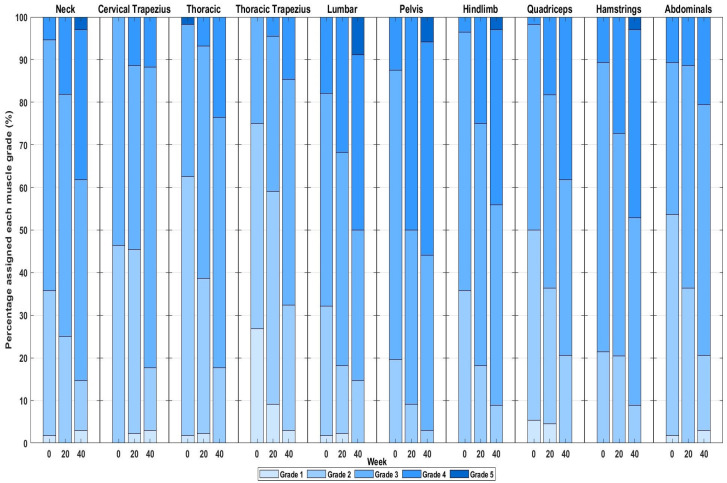
Proportion of water treadmill horses assigned muscle development grades 1–5 (Azure = 1; light blue = 2; medium blue = 3; dark blue = 4; royal blue = 5) at weeks 0, 20 and 40.

**Figure 5:**
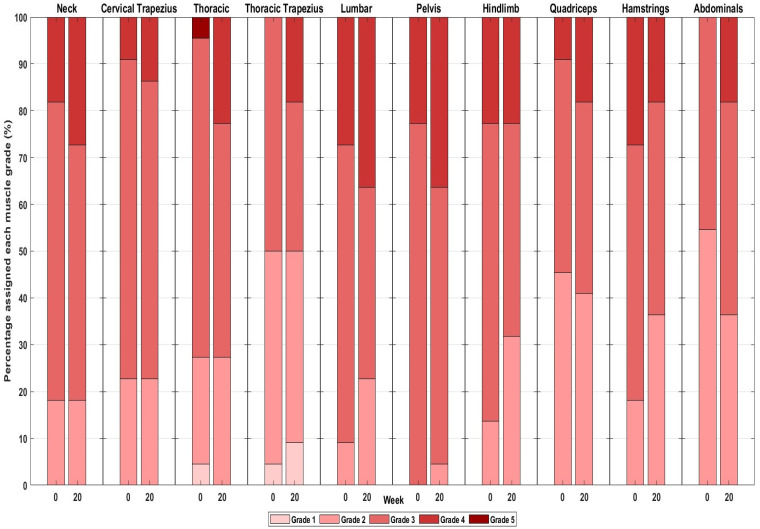
Proportion of control horses assigned muscle development grades 1–5 (Coral = 1; light red = 2; medium red = 3; dark red = 4; cranberry = 5) at weeks 0 and 20.

Between weeks 0 and 20, 11 WTH horses were lost for reasons not related to the study (unavailable to attend the week-20 testing (*n* = 6), skin disease such that they were unable to use the WT (*n* = 2), lameness (*n* = 3)), resulting in 44 WTH horses completing the 20-week period. A further 10 WTH horses were lost for reasons unrelated to the study (unavailable to attend the week-40 testing (*n* = 7), lameness (*n* = 3)), resulting in 34 horses completing the 40-week period. Between weeks 0 and 20, five Group-C horses were lost for reasons unrelated to the study (sold and moved yard (*n* = 1), lameness (*n* = 4)), resulting in 23 horses completing the 20-week period. The frequency of scores 1–5 for each muscle region is depicted in Figure 4 and Figure 5 for the WT-trained group (at weeks 0, 20 and 40) and control group (at weeks 0 and 20 only), respectively.

**Figure 6:**
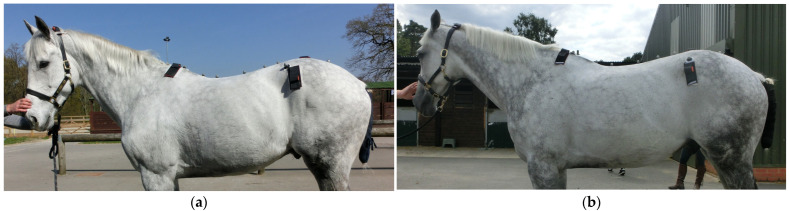
This is the same horse at week 0 (**a**) and at week 20 (**b**). Development of the throacic region increased to Grade 3 from Grade 2, thoracic trapezius increased to Grade 2 from Grade 1, lumbar region increased to Grade 4 from Grade 3, pelvis increased to Grade 4 from Grade 3, hindlimb abductor and adductor increased to Grade 3 from Grade 2 and hamstring musculature increased to Grade 4 from Grade 3 at week 20 compared to week 0.

In the WTH horses, MD of the neck, pelvis and hindlimbs significantly increased at week 20 compared to week 0 (Table 2 and Table 3). Figure 6 is composed of images of the same horse at weeks 0 and 20, where clear improvements in the development of the thoracic region, thoracic trapezius, lumbar, pelvic, hindlimb abductor and adductor and hamstring musculature can be observed. At week 40, significantly greater MD was seen for the neck, thoracic region, thoracic trapezius, lumbar region, pelvis, hindlimbs, quadriceps and hamstrings compared to week 0 (Table 2 and Table 3). Only MD of the thoracic region increased between weeks 20 and 40 (*p* ≤ 0.001 for all) (Table 2 and Table 3). In the control horses, no significant differences were seen in any region after 20 weeks (Table 2 and Table 3).

The median total BCS was not significantly different between weeks (*p* = 0.029) for Group-WTH horses (Table 4). In Group-C control horses, the median total BCS was significantly lower at week 20 compared to week 0 (*p* ≤ 0.001) (Table 4). Week-40 data was not available.

The multivariable model showed that Group-WTH horses had a statistically significantly greater total MD score (*p* ˂ 0.0001) compared to the controls at weeks 0 and 20. Horses competing at an elite level had a significantly greater total MD score than horses competing at a non-elite level (*p* = 0.009), irrespective of discipline. There was no significant difference in the total MD score between horses competing at a non-elite level and general-purpose horses. Eventing horses had a significantly lower total MD score than dressage horses (*p* = 0.013), irrespective of competition level. No other significant differences between disciplines were identified.

For Group-WTH horses, the results of the one-way ANOVA indicated that between weeks 0 and 20, using the WT more than once a week, compared to every two weeks, significantly increased hindlimb adductor and abductor and hamstring MD scores (*p* = 0.017 and *p* = 0.010, respectively). Frequency of WT usage did not significantly change the MD score for any location between weeks 20 and 40. The amount of time that the horses had been using the WT at the start of the study had no significant effect on MD.

## 4. Discussion

The objectives of this study were to describe changes in MD over a 40-week period at 20-week intervals in sport horses that use WT in their regular training regime and in work-matched control horses that do not use the WT. It was hypothesised that horses that incorporate WT exercise as part of their training regime will have greater development of the epaxial musculature and the hindlimbs than horses that do not use WT, and the results support this (see Table 2). The WT-trained horses did not show an increase in MD scores of the cervical trapezius and abdominal muscles between 0 and 40 weeks. The cervical trapezius contributes to advancement and lifting of the scapula [36]. The lack of change in the cervical trapezius was unexpected as more effort is needed to advance the scapula when walking in water. In contrast to our findings, de Meeûs d’Argenteuil et al. [28] reported significant increases in diameter of the cervical trapezius in response to eight weeks of WT training in similar-depth water, i.e., low depth, and at similar speed, but the horses in their study were training exclusively on the WT. The lack of change in abdominals is perhaps not surprising, given the gait used for training (walk), as greater surface electromyography measurements show greater activity in trot than in walk [37].

The second hypothesis was that the majority of the changes in MD would be evident between weeks 0 and 20 compared to weeks 20 and 40. WT exercise is associated with characteristic movement patterns in response to the presence of water, and so it was hypothesised that during the latter half of the 40-week training period observed, little to no muscle adaptation would occur, even when accounting for differences in experience of WT exercise prior to week 0, which varied from just four sessions to over 6 months of experience. In contrast, the horses studied by de Meeûs d’Argenteuil et al. [28] had two weeks of acclimation prior to the eight weeks of training. Rather surprisingly, the amount of time that the horses had been using the WT at the start of the study had no significant effect on MD. One explanation for this is that owners attended the WT venue more regularly during the course of the study than they would otherwise have done in an effort to be compliant with the study protocol. It is, of course, also possible that the training programmes outside of the WT sessions also had an influence on MD. Owners were encouraged to keep training diaries to note their horse’s regular weekly workload, but since neither the intensity nor the duration of the training programmes in addition to WT exercise could be captured, it was not possible to determine whether horses undergoing regular WT sessions did so in addition to, or as a replacement for, another type of training session, such as hacking or schooling. It is possible, therefore, that owners of horses within the WT group simply carried out a greater volume of total exercise per week.

In the Group-WTH horses, more significant MD changes were observed in the first 20-week period (i.e., neck, pelvis, hindlimb abductors and adductors) compared to the second 20-week period (i.e., thoracic region), where no significant changes were observed, supporting the second hypothesis. All the other changes (thoracic trapezius, lumbar region, quadriceps and hamstrings) must have exhibited smaller, gradual changes between 0 and 40 weeks. Regardless of previous experience, WT-trained horses only showed significant MD increase in the thoracic region between 20 and 40 weeks. Within a 20-week period, the minimum number of WT sessions carried out was 10 (for horses going on once every two weeks) and the maximum was 20 (for horses going on once a week). To give some context for a comparison between this study and those of others, de Meeûs d’Argenteuil et al. [28] saw muscle diameter changes following eight weeks of five-day-a-week WT training, i.e., 40 sessions, and Fair et al. [26] saw such changes following 24 WT sessions over four weeks. In the current study, the only muscle regions significantly associated with frequency of WT sessions (and only between weeks 0 and 20) were the hindlimb adductors and abductors and hamstring muscles, but not the epaxial muscles forming the ‘topline’ or the quadriceps. This may have an application in practice, whereby if hindlimb stability is required, WT session frequency of at least once a week could be considered.

Changes in MD should be considered alongside changes in the BCS. Between 0, 20 and 40 weeks, the WT horses showed no significant changes in the BCS, whilst the control horses showed a significant decrease in the total BCS from a grade of 6/9 to 5/9. Therefore, it seems unlikely that BCS changes alone account for the changes in MD scores over time since they do not reflect the changes in MD scores seen between the two groups and time points. The results indicated that horses competing at an elite level had a significantly greater total MD score than horses competing at a non-elite level, irrespective of discipline. This may be because horses working at an elite level have greater MD through cumulative training required to achieve an elite level or because the type of exercise required at an elite level is different from that at a lower level. It has been reported that horses competing at elite and low levels are at risk of different types of injury, which would support a difference in exercise type [38]. The significantly lower MD score in eventers compared with dressage horses may relate to different types of training required between the two sport disciplines. Dressage is a power sport, including many turns, circles and specific movements within a fixed arena area, while eventing incorporates straighter lines and high-heart-rate galloping exercise, so a difference in MD is not surprising. It has been shown that horses competing in dressage and eventing have different patterns of injury type, which would support differences in training and muscle use [38,39,40].

### Limitations

Ideally, all horses would have been naïve to WT exercise at week 0, but this would have been likely to necessitate a smaller sample. The assessors were aware of whether horses were part of the WTH or control groups and at which stage of the study horses were being evaluated; however, they were blinded to the grading from the previous assessment. MD was graded using a subjective scale, although it has been developed and applied by others [41]. Using an objective method, such as thoracolumbar dimensions obtained with a flexible curve ruler, may have led to different results, but alteration of thoracolumbar dimensions is affected by posture which can change before, within and after an exercise session, so a grading of MD was considered a better measure of long-term change in muscle development. There was a slightly higher proportion of dressage horses in the control group compared to the WTH group (75% vs. 60%). Breed effects could have affected MD; however, this was not assessed in the statistical analysis due to small numbers of the different breeds. Training was not standardised between horses and could have had an effect on MD. It is possible that water depth during the WT training sessions may have affected MD. Over the 40-week period, for some horses the water depth during the WT training sessions increased and for others it did not. However, they were all exercised in what is considered low water. The loss of statistical analysis for the controls at week 40 due to the small sample size is a further limitation, but was out of the study’s control due to the global pandemic movement restrictions preventing reassessment.

## 5. Conclusions

The findings of this study indicate that using WT exercise as part of a sport horse training programme increases MD compared to overground training only, particularly for musculature that supports movement patterns seen during WT exercise. This suggests that WT exercise may be beneficial for development of the hindlimb and thoracolumbosacral epaxial musculature.

## Figures and Tables

**Table 1:** Gender, height, breed and discipline distribution for 55 horses that used the water treadmill (Group WTH) and 28 control horses (Group C) that did not use a WT as part of their training regime at week 0 of the study. *n* = number; TB = Thoroughbred.

Feature		Group WTH % (*n*)	Group C % (*n*)
Gender	Mare	18 (10)	21 (6)
	Gelding	82 (45)	75 (21)
	Stallion	0	4 (1)
Height (cms)	<150	3 (2)	0
	150–158	13 (7)	29 (8)
	159–168	40 (22)	32 (9)
	169–178	31 (17)	25 (7)
	179–188	13 (7)	14 (4)
Breed	Warmblood	55 (30)	54 (15)
	TB and TB cross	32 (18)	28 (8)
	Other	13 (7)	18 (5)
Discipline	Dressage	60 (32)	75 (21)
	Showjumping	11 (6)	7 (2)
	Eventing	19 (11)	7 (2)
	General purpose	10 (5)	11 (3)

**Table 2:** Median and range of muscle development score for horses that use the water treadmill (Group WTH) and control horses that do not use a water treadmill as part of their training regime at week 0, week 20 and week 40 of the study. *n* = number of horses; * = significant difference between week 0 and week 20; ∞ = significant difference between week 20 and week 40; × = significant difference between week 20 and week 40.

	Water Treadmill Horses			Control Horses	
Location	Week 0 Median (Range) (*n* = 55)	Week 20 Median (Range) (*n* = 44)	Week 40 Median (Range) (*n* = 34)	Week 0 Median (Range) (*n* = 28)	Week 20 Median (Range) (*n* = 23)
Neck	3 (1–4) *	3 (2–4) * ×	3 (1–5) ×	3 (2–4)	3 (2–4)
Cervical trapezius	3 (2–3)	3 (1–4)	3 (1–4)	3 (2–4)	3 (2–4)
Thoracic region	2 (1–5)	3 (1–4) ∞ ×	3 (2–4) ∞ ×	3 (2–3)	3 (2–4)
Thoracic trapezius	2 (1–3)	2 (1–4) ×	3 (1–4) ×	3 (1–4)	2 (1–4)
Lumbar region	3 (1–4)	3 (1–4) ×	3 (2–5) ×	3 (2–4)	3 (2–4)
Pelvis	3 (2–4) *	4 (2–4) * ×	4 (2–4) ×	3 (3–4)	3 (2–4)
Hindlimb	3 (2–4) *	3 (2–4) * ×	3 (2–5) ×	3 (2–4)	3 (2–4)
Quadriceps	3 (1–3)	3 (0.5–4) ×	3 (2–4) ×	2 (2–4)	2 (2–4)
Hamstrings	3 (2–4)	3 (2–4) ×	3 (2–5) ×	3 (2–4)	3 (2–4)
Abdominals	2 (1–4)	3 (2–4)	3 (1–4)	2 (2–3)	3 (2–4)

**Table 3:** Results of the Friedman and post hoc Wilcoxon signed-rank tests for comparison of median muscle scores for horses that use the water treadmill (Group WTH) between weeks 0 and 20, and between weeks 20 and 40, and Wilcoxon signed-rank tests for control horses between weeks 0 and 20. Statistical differences seen in bold, Bonferonni-adjusted *p* value ≤ 0.001. n/a = not applicable.

Location	Water Treadmill Horses				Control Horses
	Friedman	Post hoc Test (Wilcoxon)			Wilcoxon
		0 vs. 20	0 vs. 40	20 vs. 40	0 vs. 20
Neck	<0.001	0.001	<0.001	0.012	0.521
Cervical Trapezius	0.011	n/a	n/a	n/a	0.739
Thoracic Region	<0.001	0.037	<0.001	<0.001	0.5
Thoracic Trapezius	<0.001	0.002	<0.001	0.029	0.42
Lumbar Region	<0.001	0.012	<0.001	0.02	0.317
Pelvis	<0.001	<0.001	<0.001	0.096	0.705
Hindlimb	<0.001	<0.001	<0.001	0.033	0.155
Quadriceps	<0.001	0.007	<0.001	0.012	0.76
Hamstrings	<0.001	0.046	<0.001	0.021	0.007
Abdominals	0.014	n/a	n/a	n/a	0.102

**Table 4:** Median and range of body condition score for horses that use the water treadmill (Group WTH) at week 0, week 20 and week 40 of the study and control horses that do not use a water treadmill as part of their training regime at week 0 and week 20 of the study.

	Water Treadmill Horses			Control Horses	
Location	Week 0 Median (Range) (*n* = 55)	Week 20 Median (Range) (*n* = 44)	Week 40 Median (Range) (*n* = 34)	Week 0 Median (Range) (*n* = 28)	Week 20 Median (Range) (*n* = 23)
Neck	6 (4–8)	5 (4–7)	6 (3–9)	6 (4–9)	6 (4–8)
Withers	5 (3–8)	5 (4–7)	6 (3–9)	6 (4–9)	6 (4–8)
Shoulder	6 (3–8)	5 (4–7)	6 (4–9)	6 (4–9)	5 (4–8)
Ribs	6 (3–8)	6 (4–8)	6 (4–9)	6 (4–9)	5 (4–8)
Loin	5 (3–8)	5 (4–7)	6 (4–9)	6 (4–9)	5 (4–8)
Tailhead	6 (4–8)	5 (4–7)	6 (4–9)	6 (4–9)	5 (4–8)

## Data Availability

Data are unavailable due to privacy or ethical restrictions.

## Supplementary Materials

The following supporting information can be downloaded at https://www.mdpi.com/article/10.3390/ani15162426/s1. Table S1: Muscle grading scale used for assessment.
