# Supplementary material for: Does Muscle Development of Sport Horses Using Water Treadmill Exercise as Part of a Long-Term Training Programme Differ from That of Horses Not Using Water Treadmill Exercise?
Source: Animals (Basel). 2025 Aug 19;15(16):2426. doi: 10.3390/ani15162426 (PMC12382896; doi:10.3390/ani15162426)
Supplement: Supplementary file 1 [file animals-15-02426-s001.zip › animals-3776157-supplementary.pdf]

## Grading

Grading was performed before exercise, with the horse standing square, the head and neck in a straight line, with the nose at the level of the shoulder and the horse attentive. Horses are assigned a grade based on 'best fit' of the statements given for each grade. To be assigned a specific grade a majority (at least 4) of statements should be true. If the horse fits between grades (i.e. 3/6 for two grades) then a 0.5 mark may be assigned.

## Definitions

Palpation should be done with the hand and fingertips, with the hand flat so that the finger tips do not dig in. Palpation should follow the direction of the hair and be for the entire length of the muscle in the specific region being graded. Pressure should be light, unless specified otherwise.

**Pressure**

- Light- pressure that depresses the area no more than 0.5cm
- Moderate- pressure that depresses the area between 0.5 and 1cm
- Strong- pressure that depresses the area more than 1cm

**Muscle tone**

- Weak= muscles are soft and flaccid. Provide minimal/no resistance to light pressure
- Slight= mild elastic resistance to pressure, but still quite soft when light pressure is applied
- Moderate= muscle feels firm but still has a reasonable amount of elasticity ('bounce') and the muscle can be depressed slightly when light pressure is applied but does not feel soft
- Good=muscle body feels firm but is still deformable, not stiff or rigid, when light pressure is applied.

**Muscle tension**

- Muscle or a specific area of the muscle is stiff or rigid and has no 'give' in it with application of light/moderate pressure


## Grading

Grading was performed before exercise, with the horse standing square, the head and neck in a straight line, with the nose at the level of the shoulder and the horse attentive. Horses are assigned a grade based on 'best fit' of the statements given for each grade. To be assigned a specific grade a majority (at least 4) of statements should be true. If the horse fits between grades (i.e. 3/6 for two grades) then a 0.5 mark may be assigned.

## Definitions

Palpation should be done with the hand and fingertips, with the hand flat so that the finger tips do not dig in. Palpation should follow the direction of the hair and be for the entire length of the muscle in the specific region being graded. Pressure should be light, unless specified otherwise.

**Pressure**

- Light- pressure that depresses the area no more than 0.5cm
- Moderate- pressure that depresses the area between 0.5 and 1cm
- Strong- pressure that depresses the area more than 1cm

**Muscle tone**

- Weak= muscles are soft and flaccid. Provide minimal/no resistance to light pressure
- Slight= mild elastic resistance to pressure, but still quite soft when light pressure is applied
- Moderate= muscle feels firm but still has a reasonable amount of elasticity ('bounce') and the muscle can be depressed slightly when light pressure is applied but does not feel soft
- Good=muscle body feels firm but is still deformable, not stiff or rigid, when light pressure is applied.

**Muscle tension**

- Muscle or a specific area of the muscle is stiff or rigid and has no 'give' in it with application of light/moderate pressure

| Score | 1 | 2 | 3 | 4 | 5 |
|---|---|---|---|---|---|
| Neck | Side area concave.<br><br>Top of neck narrow and easily moveable.<br><br>Vertebrae 1-5 visible and easily palpable.<br><br>Muscles have weak tone on palpation.<br><br>Muscle tension on palpation. | Side area concave or flat.<br><br>Top of neck flat but not convex and easily moveable.<br><br>Vertebrae 1-5 easily palpable but not visible.<br><br>Muscles have weak tone on palpation.<br><br>Muscle tension on palpation. | Top of neck convex through part of length only, with concave areas usually near the caudal extent.<br><br>Top of neck easily moveable.<br><br>Side of neck flat.<br><br>Ventral aspect of neck concave or flat.<br><br>C1-5 vertebrae palpable under moderate pressure.<br><br>Muscles have slight tone on palpation. | Top and side of neck convex through parts of length but not entire length.<br><br>Ventral aspect of neck concave or flat.<br><br>No vertebrae visible.<br><br>C1-3 vertebrae palpable under strong pressure.<br><br>Muscles have moderate tone on palpation. | Top and side of neck convex through entire length.<br><br>Ventral aspect of neck concave or flat.<br><br>No vertebrae visible.<br><br>C1-3 vertebrae palpable under strong pressure.<br><br>Muscles have good tone on palpation. |
| Neck – cervical trapezius | Shelf where neck meets shoulder.<br><br>Scapula relatively prominent.<br><br>Muscles underdeveloped.<br><br>Muscles have weak tone on palpation.<br><br>Muscle tension on palpation. | Shelf where neck meets shoulder.<br><br>Scapula visible but not prominent.<br><br>Muscles overdeveloped.<br><br>Muscles have weak tone on palpation.<br><br>Muscle tension on palpation. | Indentation cranial to withers.<br><br>Muscles have slight tone on palpation.<br><br>Normal pattern/shape of muscle development. | Slight indentation cranial to withers.<br><br>Muscles have moderate tone on palpation.<br><br>Normal pattern/shape of muscle development. | No indentation cranial to withers.<br><br>Muscles have good tone on palpation.<br><br>Normal pattern/shape of muscle development. |

| Score | 1 | 2 | 3 | 4 | 5 |
| --- | --- | --- | --- | --- | --- |
| Thoracic | Spinous processes (including left and right sides) visible and easily palpable.<br><br>Articulation with ribs visible and easily palpable.<br><br>Muscles markedly concave from side and caudal view, the shape appearing like a shelf rather than a smooth curve.<br><br>Muscles have weak tone on palpation.<br><br>Muscle tension on palpation.<br><br>Dipped back posture. | Spinous processes (including left and right sides) visible and easily palpable.<br><br>Articulation with ribs not easily visible, although palpable.<br><br>Muscles concave from side and caudal view.<br><br>Muscles have weak tone on palpation.<br><br>Muscle tension on palpation.<br><br>Mild dipped back posture. | Spinous processes visible.<br><br>Dorsal tips and left and right sides of the spinous processes palpable in dorsal 1-2cm.<br><br>Articulation with ribs not visible or easily palpable.<br><br>Muscles slightly concave or nearly flat from side and caudal view.<br><br>Muscles have slight tone on palpation. | Spinous processes visible at tip only (except at withers).<br><br>Spinous processes only palpable left and right side in dorsal <1cm.<br><br>Articulation with ribs not visible or palpable.<br><br>Muscles flat from side and caudal view.<br><br>Muscles have moderate tone on palpation. | Only dorsal tip of spinous processes palpable except at withers.<br><br>Articulation with ribs not visible or palpable.<br><br>Muscles level with spinous processes from side view.<br><br>Muscles convex from caudal view.<br><br>Muscles have good tone on palpation. |
| Thoracic trapezius | Muscles markedly concave from side view, the shape appearing like a shelf rather than a smooth curve.<br><br>Muscles have weak tone on palpation.<br><br>Muscle tension on palpation. | Muscles concave from side view.<br><br>Muscles have weak tone on palpation.<br><br>Muscle tension on palpation. | Muscles slightly concave or nearly flat from side view<br><br>Muscles have slight tone on palpation. | Muscles flat from side view.<br><br>Muscles have moderate tone on palpation. | Muscles level with spinous processes from side view.<br><br>Muscles have good tone on palpation. |

| Score | 1 | 2 | 3 | 4 | 5 |
|---|---|---|---|---|---|
| Lumbar | Spinous processes visible and easily palpable.<br><br>Transverse processes visible and easily palpable.<br><br>Tubera coxae prominent and visible and easily palpable. Muscles concave from side and caudal view.<br><br>Muscles have weak tone on palpation.<br><br>No muscle tightness/tension on palpation. | Spinous processes visible and easily palpable.<br><br>Transverse processes not easily visible, although palpable.<br><br>Tubera coxae prominent and visible and easily palpable.<br><br>Muscles concave from side and caudal view.<br><br>Muscles have weak tone on palpation.<br><br>Muscle tightness/tension on palpation. | Spinous processes visible.<br><br>Dorsal tips and left and right sides of the spinous processes palpable in dorsal 1-2cm.<br><br>Tubera coxae easily palpable but not prominent.<br><br>Muscles slightly concave or flat from caudal view.<br><br>Muscles have moderate tone on palpation.<br><br>Muscle tightness/tension on palpation. | Spinous processes visible at tip only, and only palpable left and right side in dorsal <1cm.<br><br>Transverse processes not palpable.<br><br>Tubera coxae palpable but seen only as a smooth convex region.<br><br>Muscles flat from side and caudal view.<br><br>Muscles have moderate tone on palpation.<br><br>Minimal muscle tightness. | Only dorsal tip of spinous processes palpable.<br><br>Transverse processes not palpable.<br><br>Tubera coxae hidden under muscles and only seen as smooth convex region.<br><br>Muscles convex from side and caudal view<br><br>Muscles have good tone on palpation.<br><br>No muscle tightness. |
| Pelvic | Muscles deeply concave with a shelf-like appearance from caudal and side view.<br><br>Tubera sacrale prominent – easily visible and lateral aspect visible and palpable.<br><br>Tubera coxae prominent, visible and easily palpable.<br><br>Sacrum and/or caudal (coccygeal) vertebrae definable and prominent.<br><br>Muscles have weak tone on palpation.<br><br>Muscle tension on palpation. | Muscles concave in a smooth curve from caudal and side view.<br><br>Tubera sacrale prominent – easily visible and lateral aspect palpable but not visible.<br><br>Tubera coxae prominent, visible and easily palpable.<br><br>Sacrum and or/caudal (coccygeal) vertebrae not definable.<br><br>Muscles have weak tone on palpation.<br><br>Muscle tension on palpation. | Muscles flat from side and caudal view.<br><br>Tubera sacrale prominent – easily visible and dorsal part of lateral aspect palpable.<br><br>Tubera coxae visible and palpable but not prominent.<br><br>Sacrum and or/caudal (coccygeal) vertebrae not definable.<br><br>Muscles slightly toned on palpation. | Muscles convex from caudal and side view.<br><br>Tips of tubera sacrale palpable only.<br><br>Tubera coxae palpable but seen only as a smooth convex region.<br><br>Sacrum and or/caudal (coccygeal) vertebrae not definable.<br><br>Muscles have moderate tone on palpation.<br><br>No muscle tightness. | Muscles convex from side and caudal view.<br><br>Tubera sacrale largely hidden under muscles and only seen as smooth convex region.<br><br>Tubera coxae largely hidden under muscles and only seen as smooth convex region.<br><br>Sacrum and or/caudal (coccygeal) vertebrae not definable.<br><br>Muscles have good tone on palpation.<br><br>No muscle tightness. |
| Score | 1 | 2 | 3 | 4 | 5 |

| | | | | | |
|---|---|---|---|---|---|
| Hindlimbs – medial/lateral | Muscles concave from caudal view (L= biceps femoris, vastus lateralis region; M = adductors).<br><br>Muscle tension on palpation. | Muscles concave from caudal view.<br><br>Muscles have slight tone on palpation.<br><br>Muscle tightness on palpation. | Muscles flat from caudal although isolated muscles may be concave.<br><br>Muscles have slight tone on palpation. | Muscles convex from caudal view.<br><br>Muscles moderately toned on palpation. | Muscles convex from caudal view.<br><br>Muscles have good tone on palpation. |
| Hindlimbs – quadriceps | Muscles concave from caudal view (vastus lateralis region).<br><br>Greater trochanter of femur prominent.<br><br>Muscles have weak tone on palpation.<br><br>Muscle flaccid on palpation. | Muscles concave caudal view.<br><br>Greater trochanter of femur visible but not prominent.<br><br>Muscles have weak tone on palpation.<br><br>Muscle flaccid on palpation. | Muscles flat from caudal view.<br><br>Greater trochanter of femur not easily visible but palpable with moderate pressure.<br><br>Muscles have slight tone on palpation. | Muscles convex from caudal view.<br><br>Greater trochanter of femur palpable with strong pressure.<br><br>Muscles moderately toned on palpation. | Muscles convex from caudal view.<br><br>Greater trochanter of femur requires strong pressure to palpate.<br><br>Muscles have good tone on palpation. |
| Hindlimbs – hamstrings | Muscles concave from side view (semimembranosis and semitendonosis region) and caudal view (biceps femoris region).<br><br>Tubera ischii visible and easily palpable.<br><br>Muscles have weak tone on palpation.<br><br>Muscles tight on palpation. | Muscles concave from side and/or caudal view. If only one is concave the other is flat.<br><br>Tubera ischii visible and easily palpable.<br><br>Muscles have weak tone on palpation.<br><br>Muscles tight on palpation. | Muscles flat from caudal and side view although isolated muscles may be concave.<br><br>Tubera ischii cannot be visualised easily but is easily palpable.<br><br>Muscles have slight tone on palpation.<br><br>Muscles tight on palpation. | Muscles convex from side or caudal view but not both, with other being flat.<br><br>Tubera ischii not obviously visible but palpable with moderate pressure<br><br>Muscles moderately toned on palpation.<br><br>Slight muscles tightness. | Muscles convex from side and caudal view.<br><br>Tubera ischii not visible and requires strong pressure to palpate.<br><br>Muscles have good tone on palpation.<br><br>No muscle tightness. |

| | | | | | |
|---|---|---|---|---|---|
| Abdominal | Sagging appearance to abdomen.<br><br>Abdomen appears to hang below the level of the sternum.<br><br>Dipped back.<br><br>Muscles have weak tone on palpation.<br><br>Abdomen easily moves on pressure. | Abdomen lacks shape but does not have a marked sagging appearance.<br><br>Abdomen hangs slightly lower than sternum<br><br>Moderate extension of back.<br><br>Muscles have slight tone on palpation.<br><br>Abdomen easily moves on pressure. | Abdomen is more cylindrical than sagging in shape.<br><br>Abdomen appears to be at a similar level to the sternum.<br><br>Slight extension of back.<br><br>Muscles have moderate tone on palpation.<br><br>Slight resistance to movement on pressure. | Abdomen is cylindrical in shape with minimal dropping of the ventral aspect.<br><br>Abdomen held approximately at level of sternum.<br><br>Flat back.<br><br>Muscles have moderate to good tone on palpation.<br><br>Moderate resistance to movement on pressure. | Abdomen is cylindrical in shape.<br><br>Abdomen tends to be held above sternal level.<br><br>Flat back or slight flexion.<br><br>Muscles have good tone on palpation.<br><br>Marked resistance to movement on pressure. |
